# Experience of Loneliness Among Middle-Aged Hemodialysis Patients: Qualitative Study

**DOI:** 10.1155/jonm/1013725

**Published:** 2025-05-01

**Authors:** Daun Jeong, Youn Joo Choi, Sohyune Sok

**Affiliations:** ^1^Kangbuk Samsung Hospital, Seoul, Republic of Korea; ^2^Department of Nursing, Graduate School, Kyung Hee University, Seoul, Republic of Korea; ^3^College of Nursing Science, Kyung Hee University, Seoul, Republic of Korea

**Keywords:** experience, hemodialysis, loneliness, middle-aged, phenomenology

## Abstract

**Aim:** To phenomenologically explore the subjective experience of loneliness among middle-aged hemodialysis patients.

**Background:** In South Korea, the number of middle-aged hemodialysis patients is steadily increasing, and qualitative research on the loneliness they experience is needed to understand them and develop nursing management strategies.

**Methods:** A qualitative study using the phenomenological research method of Giorgi was employed. Participants were 11 patients aged 40–64, receiving regular hemodialysis for over 3 months at a hemodialysis treatment facility in Seoul, South Korea. Data were collected from June 2021 to February, 2022, and they were in-depth interviewed until data saturation.

**Results:** The seven components derived from the study results were “The loneliness felt in a life tied to dialysis like shackles,” “The sorrow and loneliness of my irretrievable life,” “Helplessness in death and isolation at the edge of life,” “Living everyday wrapped in solitude,” “Complex emotions and alienation within the family,” “Lonely life in the shadow of illness and societal prejudice,” and “Struggling to break free from the abyss of loneliness.” Also, 20 subcomponents were derived.

**Implications for Nursing Management:** Despite facing the negative aspects of loneliness associated with illness and treatment, middle-aged hemodialysis patients accept loneliness as a meaningful adaptive experience. This repetitive phenomenon throughout the life cycle of lifelong dialysis patients suggests a continuous process. In nursing management, nurses or nursing managers need to pay attention to the results of this study for deep understanding of middle-aged hemodialysis patients and qualitative nursing management. Based on the results of this study, nursing management strategies for them can be developed.

## 1. Introduction

End-stage renal disease (ESRD) is a condition in which kidney function is irreversibly damaged, requiring renal replacement therapies. Hemodialysis is used as one of the important treatments for patients to maintain their daily lives and reduce the risk of complications [1]. Currently, the number of hemodialysis patients is steadily increasing in Korea, accounting for 81% of renal replacement therapies [2].

Patients receiving hemodialysis experience major lifestyle changes due to the need to regularly undergo dialysis for four to five hours, twice or thrice weekly [3]. These treatments do not solve the fundamental problem, so patients still experience physical and psychological difficulties, depression, limitations in social activities, and role changes [4–6]. Particularly, middle-aged patients face a dual burden of fulfilling their roles as both patients and family members. As patients, they must actively participate in their treatment regimen, follow medical recommendations, and manage their health conditions [7]. Simultaneously, as family members, they are expected to provide financial stability, support family members emotionally, and maintain household responsibilities [8]. The need to balance these roles increases psychological stress, especially in middle age, when financial responsibilities and social expectations are at their peak [9, 10].

Loneliness is one of the high-level problems experienced by hemodialysis patients. Middle-aged patients may especially have frequent suicidal thoughts due to the lack of social support and relationships [11]. Lack of social support and relationships can cause depression and trigger suicidal thoughts, which can reduce the patients' psychological health as well as immunity, making them more vulnerable to disease [11, 12]. According to the National Institute of Korean Language's Standard Korean Language Dictionary [13], *loneliness* is defined as “being very lonely and forlorn, as if one is alone in the world.” Humans need interaction to survive and establish a sense of identity, but when they lack satisfactory relationships with others, they have the painful experience of loneliness [14]. Loneliness can be interpreted as maladaptation due to the absence of social relationships or as a negative experience in social relationships, and many scholars emphasize maladaptation due to the lack of relationships in their psychological research, explaining loneliness as a cause of various psychological disorders [15–17]. However, loneliness is considered an essential element for spiritual growth, change of ideas, and new enlightenment in philosophy, religion, and art. Since it is realistically difficult to satisfy all social needs, an appropriate experience of loneliness is considered a vital element for adaptation and independence [18–20].

Middle-aged hemodialysis patients experience loneliness due to multiple factors, including physical limitations, social isolation, economic hardship, shifting family roles, and a lack of emotional support [11, 12]. The ongoing necessity of dialysis makes it difficult for them to maintain employment and engage in regular social activities, leading to estrangement from their colleagues and community [9, 10]. Economic difficulties often arise due to reduced work capacity, further compounding stress and anxiety [21, 22]. Additionally, these patients may perceive themselves as burdens to their families, leading them to suppress their emotions rather than seek support. Such factors can contribute to a heightened sense of loneliness, increased psychological distress, and a reduced quality of life (QOL) [15–17].

Loneliness experienced in restrictive treatment situations, such as hemodialysis in middle age, a period of developmental tasks, significantly affects psychological, physical, and social health, which can be carried over into old age [21–23]. During middle age (40–64 years), individuals are expected to maintain a stable career, achieve financial independence, and foster strong relationships within their families and communities [7, 8]. They also begin preparing for a healthy retirement by adopting proper health behaviors. However, the demands of hemodialysis disrupt these developmental tasks, often resulting in financial instability, workforce disengagement, and increased dependence on caregivers [9, 10]. These challenges not only diminish their current well-being but also negatively impact their long-term health, increasing vulnerability in old age [21, 22].

The researcher developed an interest in this study based on clinical experience, believing that exploring the essence of hemodialysis patients' experiences during treatment is crucial for improving positive treatment outcomes and enhancing their QOL. Through clinical practice, the researcher observed that middle-aged hemodialysis patients face a unique set of challenges beyond medical treatment, including difficulties in fulfilling their social and familial roles. Understanding these experiences in depth is essential for developing patient-centered nursing interventions that support both their physical and psychological well-being. Thus, it is necessary to explore in depth the loneliness that middle-aged patients experience while receiving hemodialysis and identify the unique experiences they have while receiving hemodialysis and the nursing interventions they need.

Most previous studies focus on social support, meaning of life, depression, and family burden of elderly hemodialysis patients [22–24]. However, there is a lack of research on psychological and social aspects targeting middle-aged patients, and in particular, there has been a limitation in the in-depth exploration of the loneliness they experience.

This study aims to explore the essence of loneliness experienced by middle-aged hemodialysis patients using Giorgi's descriptive phenomenology [25], with the goal of identifying the specific challenges they face within their unique circumstances and the support they require. Through this, the study seeks to provide insights relevant to nursing practice and contribute to the development of patient-centered nursing strategies. The research question for this is, “What is the experience of loneliness among middle-aged patients receiving hemodialysis?”

## 2. Methods

### 2.1. Design, Sample, and Settings

This study was a qualitative study using the phenomenological research method of the author in [25], using data collected through in-depth interviews to explore the meaning and essential structure of loneliness experienced by middle-aged hemodialysis patients. Based on the researcher's clinical experience and previous studies, understanding the essence of patients' experiences during the treatment process is crucial in promoting positive treatment outcomes and improving their QOL. Therefore, phenomenological qualitative research, which focuses on exploring the essence of lived experiences, was employed in this study. Participants in this study were middle-aged patients aged 40 to 64 years who were receiving regular hemodialysis at least three times a week for at least three months at a hemodialysis treatment center in Seoul. The study was limited to patients without serious mental illness, who were able to communicate, understand, and respond to interview questions, and who fully understood the purpose of the study and voluntarily agreed to participate in the study. The inclusion criteria were set to include physically and emotionally stable patients. In particular, patients who had been on hemodialysis for less than 3 months were excluded from the study, as patients in the early stages of dialysis are likely to experience greater emotional anxiety and physical distress during the disease adjustment process, which may differ from the loneliness experience we aim to address in this study [5, 6]. Participants were interviewed until data saturation, and a total of 11 middle-aged hemodialysis patients were finally included in the study.

The recruitment process for participants was conducted through direct contact. The researcher approached hemodialysis patients at the hospital, providing a detailed explanation of the study's purpose, procedures, and measures for the protection of personal information and anonymity. Written informed consent was obtained from patients who agreed to participate, and each participant voluntarily enrolled in the study after receiving a thorough explanation of the research procedures.

Furthermore, loneliness was not explicitly set as an inclusion criterion in this study. While the research aimed to explore the essence of loneliness experienced by middle-aged hemodialysis patients, it did not exclusively target patients who had clearly experienced loneliness. Instead, middle-aged patients who had been receiving hemodialysis for more than 3 months and who understood the study's objectives were selected. As a result, loneliness naturally emerged as a phenomenon within the dialysis experiences of the participants, rather than being a predetermined inclusion criterion.

### 2.2. Data Collection

The study's data, collected through face-to-face semistructured interviews with 11 middle-aged hemodialysis patients, were gathered from June 2021 to February 2022. After classifying the participants' responses to the in-depth interviews into common themes or subcomponents and sufficient descriptions to explain the relationships between the components being presented, saturation, where no new themes were developed, was reached after 13 interviews. In phenomenological qualitative research, interviews were conducted until data saturation was reached, meaning that no new experiences or themes emerged from participants' responses. Saturation ensured that the essence of the participants' experiences had been fully explored [26]. Therefore, interviews were conducted as needed until saturation was achieved, requiring one to two interviews per participant. The second interview provided an opportunity to verify and expand upon the information shared in the first session, ensuring that all aspects of the participants' loneliness experiences were thoroughly captured. Additionally, any missing details or ambiguities in the initial responses were addressed through follow-up questions during the second interview. The maximum number of interviews for each participant was two. Each interview took about one to 2 hours, and the location and date of the interview were selected after considering the participants' physical vulnerability and convenience. Thus, interviews were conducted before starting dialysis or on a day without dialysis in a counseling room with a quiet atmosphere located within the facility.

In this study, open-ended and semistructured interview questions were utilized to maintain objectivity while deeply exploring the experiences of loneliness among middle-aged hemodialysis patients. The researcher, drawing on 14 years of rich clinical experience as a hemodialysis nurse, formulated the interview questions to encourage participants to freely describe their experiences. No separate pilot test was conducted, and caution was taken to avoid leading or suggesting answers during the interview process.

The interviews were designed to align with the study's purpose of deeply understanding the lives and experiences of loneliness among middle-aged hemodialysis patients. Key questions included the following: “How do you feel and think about your life while undergoing hemodialysis?”, “What does the loneliness you experience while receiving hemodialysis mean to you?”, “In what situations or moments do you feel the most lonely?”, “How has hemodialysis affected your family or social relationships?”, “What efforts have you made to overcome or alleviate loneliness?”, and “Have there been any changes in the meaning or perspective of life as you experience loneliness?”. Additionally, to conclude the interview, questions such as “Is there anything more you would like to share about your experience of loneliness while undergoing hemodialysis?” were asked to allow participants to share additional experiences.

To prevent data omission and ensure reliability during the in-depth interviews, digital recorders, smartphone recorders, and field notes were used with participants' consent. Additionally, the researcher took care not to induce or suggest responses. To enhance data accuracy, the recorded content was transcribed verbatim in the participants' own language, and any omissions were addressed through follow-up questions during the second interview.

None of the participants refused or withdrew from the study. This outcome seems to be a result of the researcher's flexibility in adjusting the interview schedules according to the participants' availability and physical condition, ensuring they did not feel psychological pressure.

### 2.3. Data Analysis

The collected data were analyzed using Giorgi's [25] phenomenological analysis method, with data analysis conducted concurrently with data collection. The first step involved interpreting the essence of the respondents' statements by reading the participants' descriptions in their entirety, maintaining a natural attitude. To grasp the overall meaning, the researcher repeatedly read through the responses. In the second step, the transcribed content was divided into “meaning units” by carefully identifying shifts in the experience of loneliness, marked with a diagonal line (/). Meaning units were divided into paragraphs rather than sentences to account for context and were organized based on intentionality, the central theme of phenomenology, and the researcher's nursing perspective. This process resulted in 485 meaning units from 11 participants. The third step involved transforming everyday expressions into academic terms. The meaning units were compared, consolidated, and converted into appropriate academic terms through reflection and imaginative transformation. When no established academic term was available, common language was used from a phenomenological standpoint. Finally, the transformed meaning units were integrated into a structure. The researcher derived components from the meaning units and employed imaginative variation to determine the general structure of the experience. Constant consideration of exceptional cases allowed the researcher to uncover the essence of the loneliness phenomenon. To ensure validity, the researcher revisited the original data, re-reading and reflecting continuously. This process yielded 65 academic transformations, 20 subcomponents, and seven components.

Data coding was conducted solely by the researcher. Drawing on 14 years of clinical experience and expertise in phenomenological research methods, the researcher analyzed the data. Microsoft Word and Excel were used to transcribe the handwritten notes, classify them into meaning units, and perform the coding process. All data were stored on a password-protected personal computer, with a dual security system in place to maintain confidentiality.

### 2.4. Ensuring the Validity of the Study

To ensure the rigor of this study, the evaluation criteria proposed by Lincoln and Guba [26] were applied. The researcher conducted a repetitive process of comparing the findings with the participants' original statements through continuous reflection and questioning to ensure the accuracy of their perceptions and experiences. The plausibility of the analysis was enhanced by linking the results with meanings derived from various data sources. The validity of the findings was confirmed by presenting the analysis to some participants for review and receiving feedback to ensure that the results aligned with their experiences.

Due to the time and physical limitations of the participants, it was challenging to obtain feedback from all of them. Middle-aged hemodialysis patients experience physical fatigue and constraints due to treatment, making it impractical to seek feedback from everyone. Therefore, feedback was requested from select participants to sufficiently reflect various perspectives and strengthen the validity of the results. Notably, those who provided feedback were individuals who contributed significant insights to the study, thereby enhancing its reliability.

In addition, to verify the applicability of the findings, interviews were conducted continuously until no new information emerged, ensuring sufficient description of the participants' statements. The results were also presented to three middle-aged hemodialysis patients who did not participate in the study to confirm whether they resonated with the findings. To maintain consistency throughout the research, Giorgi's method was adhered to in all phases, and the sociocultural context surrounding the participants' lives and experiences was considered in exploring the meaning of their experiences. The data collection and four-step analysis procedure outlined by the author in [25] were rigorously followed to derive the results. Moreover, the researcher consciously minimized the influence of personal clinical experience and existing knowledge to avoid bias in understanding the phenomenon. Until the data collection was completed, a thorough literature review was limited, and the related theoretical content was carefully organized to prevent theoretical bias from affecting the participants' statements.

### 2.5. Researcher Preparation

The researcher has worked as a hemodialysis nurse for 14 years, gaining clinical knowledge and expertise in the field by participating in numerous dialysis-related conferences and research programs. During the doctoral program, the researcher completed coursework in qualitative research methodology and continued to enhance research skills by attending various academic conferences and workshops. The researcher focused on mastering data collection and analysis in descriptive phenomenology through targeted education and training.

### 2.6. Ethical Considerations

This study was conducted after receiving approval from the Institutional Review Board (IRB) of K Hospital (IRB File No. 2021-04-016-002). To protect the rights of the participants, the researcher provided both oral and written explanations about the purpose and procedures of the study, as well as assurances of anonymity, confidentiality, the use of recorded interviews solely for research purposes, and the participants' right to withdraw from the study at any time. The study proceeded with participants who provided written consent. Interviews were conducted in a counseling room within the hospital at a time chosen by the participants, ensuring a comfortable environment. The collected data were stored as password-protected Word files on the researcher's secured personal computer. After the interviews, participants were offered a small token of appreciation for their time and effort.

## 3. Results

The 11 participants in this study comprised two patients in their 40s, seven in their 50s, and two in their 60s, where seven were women and four were men. The average duration of dialysis varied from two to 21 years. Nine participants were married, and two were unmarried. Regarding the presence or absence of a cohabiting family member, 10 participants had a cohabiting family member and one did not have a cohabiting family member. The occupation distribution was as follows: one participant had a full-time job, three had a part-time job, two were self-employed, and the remaining participants had currently stopped working (Table 1).

### 3.1. Situational Structural Description of Study Participants

Seven components and 20 subcomponents were derived after analyzing the data using the phenomenological analysis method of the author in [25]. The components derived from the study results were “The loneliness felt in a life tied to dialysis like shackles,” “The sorrow and loneliness of my irretrievable life,” “Helplessness in death and isolation at the edge of life,” “Living everyday wrapped in solitude,” “Complex emotions and alienation within the family,” “Lonely life in the shadow of illness and societal prejudice,” and “Struggling to break free from the abyss of loneliness” (Table 2).

#### 3.1.1. The Loneliness Felt in a Life Tied to Dialysis Like Shackles

The participants felt helpless and sad not only due to time and physical limitations caused by dialysis but also due to difficulties in participating in interpersonal relationships, trapped golden years, and the life in a space called a hospital that they cannot escape from for the rest of their lives. These restrictions made them feel their lives like shackles.

##### 3.1.1.1. Deep Sense of Loss and Loneliness Stemming From the Constraints of Dialysis

As the participants continued to undergo dialysis, their physical strength rapidly decreased, and they were unable to do anything due to the dialysis schedule, so they spent time in vain and regret. They also experienced dizzying moments while experiencing emergency dialysis due to dietary restrictions and expressed sadness at not being able to enjoy food to their heart's content. They shared their experiences of isolating themselves due to time and physical limitations and giving up everything rather than overcoming life in realistic situations.

*“There are many cases we need to be choosy about food. We dialysis patients often feel swollen or short of breath when we eat even a little bit of salty food. One time, after eating watermelon in the summer, my chest was so tight that I had to go to the emergency room and get urgent dialysis. I was really scared at that time. Sometimes I think [about] why I became the kind of person who can't eat what I want… I felt empty and sad.” (Participant 4)*

*“It's true that I have to think about my body, but I also want to have a child and live a normal life like everyone else. But I have to undergo dialysis and take a lot of medicine, so I gave up on getting pregnant. Even if I give birth, I will be sick and it will be difficult to raise the child. I have no choice but to hope that it is possible in my next life.” (Participant 8)*

##### 3.1.1.2. Feeling of Ostracism due to Difficulty Participating in Social Activities Because of Illness

While undergoing dialysis, participants found it difficult to travel with family or make appointments with acquaintances. They heard news about small groups they could not participate in and felt lonely. In particular, it was difficult to participate in long-term trips with their family, so they felt sorry for missing out on precious moments with their family. Even when meeting with acquaintances, there were frequent moments where they felt left out because they could not easily make an appointment.

*“In the past, if I suggested something to do regardless of the time, everyone was willing to do it, but now, on the contrary, even if they suggest a time, I have to say No. They try to read my thoughts in that part. And these days, with the development of social media, when my friends post a picture of them hanging out while I'm lying down at [the] hospital on Fridays, receiving dialysis… I feel a little alone for a moment… Now I feel like I'm becoming what the kids say these days, “Assa (outsider). I feel like I'm becoming a loner and moving further and further away…It ended up being that kind of situation.” (Participant 11)*

##### 3.1.1.3. Endless Emptiness and Loneliness Felt While Confined in a Hospital Environment

The participants felt the emptiness of a meaningless life within the inescapable limits of the hospital and faced the hopeless reality of an incurable disease. In particular, as a patient, they experienced low status from medical staff and became more aware of their helplessness due to physical complications that occurred during treatment. In this situation, they experienced the difficulty of finding meaning in their lives as their sense of helplessness and letdowns worsened.

*“This disease is the most difficult. Coming every two days and lying still for 4 hours… This is not something you can overcome by exercising. It's not curable, so do I really need to live like this to get treatment? I think about that a lot when I'm lying down at [the] hospital. And while on dialysis, I feel like I've experienced all the physical hardships I've never experienced before. During dialysis, I suddenly wanted to go to the bathroom, so I said I'd go, but my blood pressure suddenly dropped, so they didn't let me go, saying it is dangerous… I felt miserable because there was nothing I could control with my own will.” (Participant 6)*

*“I did not become [sic] a patient because I wanted to be a patient with a disease, but I feel intimidated when I go to the hospital without realizing it. I want to be close to the people I have to see every time I get dialysis until I die… but I think I'm drawing a line. Because I'm just a patient at the hospital… that's true, but there are a lot of times when I feel sad.” (Participant 11)*

##### 3.1.1.4. Loneliness in Old Age Hindered by Reality due to Chronic Illness

The participants wanted to spend their golden years in a quiet countryside or their hometown, but the dream was hindered by the reality of having to stay near a dialysis hospital, where they had to constantly stay in distant treatment locations. In addition, due to the financial burden caused by dialysis, they had to focus on spending on raising their children, and their dreams of golden years became increasingly distant with the futility of living on dialysis.

*“In the past, I wanted to live comfortably in a place like Jeju Island or a quiet countryside, but now I'm receiving dialysis, so actually I have to continue to live near a hospital, which makes my life restricted. Even when thinking about the future, I feel like now I'm being held back because I have to live near a dialysis hospital.” (Participant 7)*

#### 3.1.2. The Sorrow and Loneliness of My Irretrievable Life

The participants felt that their current life was very different compared to their glamorous life before dialysis, and because of this, they were experiencing loneliness and lamenting about their situation not going according to their will.

##### 3.1.2.1. Longing and Loneliness for the Days Before Dialysis

Before dialysis, participants had no restrictions on fluids and diet and enjoyed traveling freely regardless of time and place. In addition, while working at a full-time job, they prepared for retirement and were economically stable. There were many times when they found it difficult to accept reality, missing the glamorous life they had before dialysis, when they were able to make all their own decisions and had no difficulty participating in various activities, studies, and self-help groups.

*“Before dialysis, it was completely different than it is now. Well, there was nothing particularly difficult. I was free to eat, travel, and learn.” (Participant 8)*

*“Before, when I was working, my aspirations for the future were a huge part of my life, but now I can't work, so I gave up. Nothing in the world is going the way I want…” (Participant 7)*

##### 3.1.2.2. Disappointment and Estrangement in a Life not Going as Planned

In the life of repeated dialysis, they felt bitter as they repeated the thought many times that they could not enjoy life as diversely as others, since the normal daily life for others became a heavy burden to them. In particular, middle-aged persons with long-life expectancy experienced sadness and isolation due to the loss of time and freedom due to dialysis. At the same time, due to the incurable disease, they continued to question the meaning of life and experienced a sense of disappointment.

*“Dialysis itself was just unfair and sad… My freedom of time disappeared regardless of my will. The time constraints are too big. So, I think this becomes truer the younger you are. I'm not that old… Even now, I can't move the way I want to, so I can't do [it] even if I want to. I am really receiving treatment diligently…It's not curable, so do I really need to live like this to get treatment? This situation is meaningless.” (Participant 10)*

##### 3.1.2.3. Solitude and Loneliness Felt Looking at Oneself Significantly Changed From the Past

The participants experienced loneliness as they faced their current shabby reality, where they were discouraged and had significantly lowered self-esteem, and saw themselves changing differently from before.

*“Now, what changed before and after receiving dialysis is a lack of confidence in physical strength and such aspects… While receiving dialysis, I have time constraints… As I started to lose confidence and things like that, I started to become passive…I couldn't take my friends' jokes as jokes and I became intimidated without even realizing it.” (Participant 11)*

#### 3.1.3. Helplessness in Death and Isolation at the Edge of Life

The participants suffered from extreme loneliness, sometimes experiencing ambivalence about maintaining or discontinuing treatment, and faced the boundary between life and death.

##### 3.1.3.1. Deep Despair and Loneliness to the Extent of Thinking About Death

When the participants thought about death, the world suddenly became dark and they felt lonely and desolate, as if they were alone. In these moments, they could not sleep, and their feelings of loneliness became even stronger. They said that sometimes they thought about death to the extreme, but they also felt worry and fear because they did not want to harm their family.

*“After dialysis, I'm having a hard time, so there are times when I lie down alone without my family and think about this and that. ‘Why am I living like this…' There are times when loneliness suddenly comes flooding back. My apartment is on the* 4^th^*floor. I said I wish I could jump out of here and die. I would be glad if I could jump and die, but what if it doesn't work? All I could think about was this… ”(Participant 1)*

##### 3.1.3.2. Existential Isolation Felt From Losing the Meaning of Life

The participants said that due to dialysis, the future was uncertain and vague and that it was difficult to find a reason to live in a reality where they had experienced only pain and discomfort for a long period. Sometimes, they even vaguely thought that they wished there was no tomorrow.

*“I didn't have the confidence to survive while on dialysis, so I even thought about trying to get through it without having* a *nephrectomy for kidney cancer. Because if I don't remove the cancer, I will die… No matter what I did, there was no hope.” (Participant 7)*

*“The life ahead of me… I don't know… Well, I have no intention of living any longer… When I wake up, I wish there was no tomorrow.” (Participant 3)*

#### 3.1.4. Living Everyday Wrapped in Solitude

The participants accepted the disease as their pain, endured it alone, and made efforts to live well with loneliness.

##### 3.1.4.1. Sense of Isolation in Illness That Must Be Endured Alone

The participants expected to receive quiet support from people around them in moments of loneliness and attempted to shake off the situation on their own. Whenever they felt alienated, they persuaded themselves that humans naturally felt lonely, and they tried to endure it alone because their life receiving hemodialysis was considered an incomprehensible pain.

*“Honestly, right now, the life I am living while receiving dialysis at this young age… Only I myself know what's inside my heart. Family too… No one…. I'm just trying to survive in this situation on my own. I have to live it.” (Participant 8)*

##### 3.1.4.2. Living Well Within the Lonely Confines of Illness

Participants carefully dream of being no longer sick, living well as they are now, even in situations where they have to undergo lifelong dialysis, helping others, and living well without being a nuisance to their families. In addition, they had the will to persevere in finding hope even amid illness through their desire to become helpful people in society by utilizing their talents.

*“Even though I am a person receiving dialysis, I can communicate with people around me, and now communication can become a job. Because I have to make money. So, I want to do something that helps through communication.” (Participant 5)*

#### 3.1.5. Complex Emotions and Alienation Within the Family

The participants experienced complex ambivalence and felt extremely lonely when they felt that their situation was not understood by their families. Moreover, during the developmental task of middle age, the participants experienced despair at the reality of not being able to help the family because it was difficult to play their role as a family member.

##### 3.1.5.1. Alienation Stemming From Mixed Feelings Toward the Family

The participants said that they wanted their families to know when they were in trouble, but on the other hand, they did not want to receive unconditional consideration as sick patients. Instead, despite the pain, they hoped for their family to watch them playing their role as the head of the family as a supporter, and they experienced ambivalence, feeling grateful and sorry for their family at the same time.

*“I was really going to die after coming home from dialysis… Why don't you know? I feel really lonely with these thoughts, and there are times when I really feel like I'm the only one who knows it…even family is useless. However, as the head of the family, I just hope for them to watch me as an unwavering supporter. It doesn't make sense, does it? (Laughs awkwardly) Those people probably didn't think about that part at all. Because I'm the only one who feels that way. I don't like it when my family only gives me consideration.” (Participant 11)*

##### 3.1.5.2. Loneliness Felt From the Inability to Share Emotions

Despite being in the same space as their families, the participants sometimes felt alone. In particular, it was hard and lonely when their families did not fully understand their difficult situation. When they felt this way, they hoped their family would understand and sympathize with them.

*“Am I the only one who is sick?… Am I the only one having a hard time?… So, is it just me who is having a hard time?… I have to live like this for the rest of my life… I just have bad days. I'm so tired that I'm dying, but when people don't know me, it's very difficult, lonely, and sad. Sometimes, I don't have to go to my in-laws' events because I‘m having a hard time after receiving dialysis, but there are some elderly people who wish I could come. Then, when I have to go even though it's hard, it's really hard on my mind and I have to read their face.” (Participant 8)*

##### 3.1.5.3. Powerlessness and Estrangement Felt From the Inability to Be Helpful as a Family Member

When performing their role as a family member was difficult, the participants experienced a sense of loss. As a parent, they should participate in their child's growth process, but they expressed regret that they could not participate in the experience due to being on dialysis. They felt sorry for their family, feeling that they were a “nuisance,” and mentioned the experience of feeling sorry and lonely because their family's life was affected by their illness. They also talked about the aspect of feeling empty, not being able to fulfill their role as a spouse, and only causing worry to their family.

*“I can't even act as a mother to my daughter… At best, they changed my dialysis schedule on the day my daughter took the college scholastic ability test, so I could go with her… Besides that, if my child has an open class, I can't attend and I can't do my duty as a mother at all… There is a limit to my time when I have to attend things I need to attend or take care of my child, and even at home, I have to give up everything on the day I receive dialysis. That's why I'm such a nuisance right now.” (Participant 5)*

*“I want to be a good husband… From my wife's point of view, I am a sick head of the family, a bit frustrated, and a husband who is not doing his role properly. How anxious she is that I might suddenly become very ill. I just feel sorry. But I tried my best, but I think I'll be disappointed if she just thinks of me as a sick husband… ”(Participant 10)*

#### 3.1.6. Lonely Life in the Shadow of Illness and Societal Prejudice

The participants always perceived themselves as weak wherever they were, and when they received help, they felt that they had become truly socially weak and experienced loneliness. In addition, due to their situation not meeting the work requirements, they experienced many limitations in their work life, experienced the feeling of being alone and isolated even when they were with people, and even isolated themselves by hiding their illness to avoid social prejudice.

##### 3.1.6.1. Continuous Sense of Exclusion Felt From Always Being Perceived as Weak

Participants always perceived themselves as weak in terms of health. In an unusual reality, there are times when they feel that their lives are meaningless because they realize they are living a different life than ordinary people. In addition, while expressing gratitude for the government's support benefits, they expressed a lonely feeling that being revealed as a disabled person while receiving help would make them truly socially vulnerable.

*“I feel like a person who is not helpful in this society called Korea. Because I don't know how to do anything, there's no one whom I can help… rather, I get help. I'm more miserable because I feel like I'm disabled… I wish my family wouldn't treat me like a patient. …(omitted)… The disability pension I receive… It's not a happy thing for me because it reveals that I'm disabled, but I get it.” (Participant 9)*

##### 3.1.6.2. Loneliness Arising From Limited Work Opportunities

The participants experienced many limitations in their work life due to dialysis. On the day they returned home from dialysis, they were physically tired and could not do anything, and there were many days they spent lying down, so they tried to find a job on days without dialysis, but their job-seeking activities were also limited due to the dialysis schedule. In addition, it was unthinkable to work full-time, and it was thought that re-employment would be difficult if it became known that the person was receiving dialysis even at a part-time job. This indicated that participants were experiencing more sadness and unpowerness due to restrictions on their work life.

*“Even if I want to have a simple part-time job, I have to miss out on Tuesday, Thursday, and Saturday. Who would hire me? There is no one hiring me. On the day I come back home after receiving dialysis, I am lying down unable to do anything, so I cannot have a job. Then, what kind of boss would hire me only for Monday, Wednesday, and Friday? Also, if they know that I am sick, wouldn't they hire me more? Indeed….” (Participant 5)*

##### 3.1.6.3. Isolation and Alienation Stemming From Societal Prejudices

The participants expressed regret over undergoing dialysis at a young age due to an illness that occurred against their will. However, those around them did not understand this and made indifferent words and actions, which made them feel hurt and sad. Due to these experiences, the participants were reluctant to communicate with others, ignored others as they did not want to explain, and disconnected themselves from others. Because of the physical changes after dialysis, they did not want to attract attention from others, so they hid themselves and tried to understand and hide their fears. In particular, as they were worried about the negative perception and social impact of the label “disabled if you are on dialysis,” they isolated themselves by not meeting new people.

*“This is a disease that came against my will… If I receive dialysis, I have to be called disabled. I hated it so much, and I was worried that it would affect my kids, and I felt sad about this situation… It doesn't help. I've never been able to properly go to the public bath with my kids. I don't like showing my arms and veins… I'm afraid that others will look at my protruding arms strangely… Also, I'm afraid that the kids will get hurt if they hear things like that because of that. That's why I don't go out as much as possible.” (Participant 9)*

##### 3.1.6.4. Loneliness and Estrangement Deepening as the Perception of Being Alone Intensifies

The participants did not let others know about their pain but smiled brightly and pretended like nothing was wrong, but inside, they lamented about their lives of pain and depression. Because of this, they felt alone and lonely.

*“To be honest, I don't look like a sick person when I go out. You know the swan? It looks elegant on the outside, right? How much do they wiggle their feet to stay afloat? The inside is rotten and cracked. But although I smile brightly without showing any signs on the outside, I am very festering on the inside… If people find out, they will feel sorry for me. I don't like that. I'm the only one who knows how lonely this is….” (Participant 5)*

#### 3.1.7. Struggling to Break Free From the Abyss of Loneliness

The participants tried to fight loneliness with the support of their families, who were the supporters of their lives, and gain strength through communication and positive diversion whenever they were lonely and challenged.

##### 3.1.7.1. Efforts to Overcome Loneliness With the Support of Family

Family helped the participants feel less alienated. The participants' family members always took care of them, comforted them when they were struggling, lonely, and lost the meaning of life, and reminded them of the meaning of life. The participants tried to escape from loneliness by making up their minds not to show their lonely and difficult feelings for the sake of their families.

*“The closest people are family. My younger brother, my younger sister, and my nephews… In the case of my eldest nephew, calls me once a day even though he is busy, and he is the biggest help to me. When we're together, I don't have to think about anything.” (Participant 6)*

##### 3.1.7.2. Attempts to Reduce Loneliness Through Meaningful Communication and New Activities

When the participants were lonely or having a hard time, they tried to forget their feelings in a space with other people or to deal with the situation by talking on the phone with family or friends. They also felt empathy and comfort within the community of people in the same situation undergoing hemodialysis. They tried various methods to forget loneliness, such as listening to music, walking, reading, and watching people.

*“There is a cafe where people gather on the Internet. About 7 of us gather together, and we all receive dialysis. When I talk to those friends, honestly, no matter what I say, they understand everything. Because it's about things that happened during dialysis… In fact, it really helped me a lot. Maybe that's why I didn't get depressed. I can tell them things that I can't tell my family. I can only tell them. Because they know what the situation is. Even if I just say this, they immediately say, ‘That?' So, I get comforted I'm not the only one having this hard time…'” (Participant 8)*

*“Just listening to music and walking alone… I go to the crowded Han River, listen to music, smoke a cigarette, watch people passing by, and go back and forth without thinking… Now, if I think about this and that, I feel like it will become more difficult… I just think it's the most comfortable. Just without thinking… just looking at people passing by… I refresh myself while watching people.” (Participant 11)*

### 3.2. General Structure of Loneliness Experience in Middle-Aged Hemodialysis Patients

Participants in this study experienced a sense of loneliness and loss amidst the limitations and difficulties caused by living on dialysis. Living in the hospital brought about loneliness and sadness, and anxiety about old age emerged due to high treatment costs and financial constraints. The repetition of dialysis felt like a shackle in their lives, and they experienced loneliness as a result. Additionally, the participants recognize loneliness as a process of independence and adaptation and seek to live strong despite their illness. However, within realistic constraints, they realize the limits of daily life, experience a sense of loss about their situation that is different from before, and consequently, it becomes difficult to find the meaning of life, and sometimes, they experience ambivalence about death internally.

In their relationships with their family, they had the desire to fulfill their role, but also, they felt sad for not being able to provide utility to their family and experienced loneliness as they were unable to convey their honest feelings. This lack of communication with family leads to deeper loneliness, coupled with isolation due to social prejudice.

The disability caused by hemodialysis increases the fear of social prejudice, and restrictions and difficulties at work further amplify the loneliness of being alone. These middle-aged patients attempt to escape loneliness through family support and try to overcome loneliness through communication and diversion.

This experience of loneliness consists of intrapersonal, individual-family, individual-society, and individual-family-society relational aspects. This experience of loneliness, which occurs simultaneously within the continuity of life, changes depending on time and context, so patients try to accept it not only negatively but also positively.

## 4. Discussion

It was observed that middle-aged hemodialysis patients experience loneliness in various aspects of a treatment situation that leads to death and accept this as a meaningful experience of adaptation. This experience is repeated cyclically throughout their lives and has similar characteristics to the loneliness experience of the elderly [27]. However, it was different from the loneliness of single-room dwellers shown in a previous study in the context of time flow [28].

Middle-aged hemodialysis patients who experience loneliness are especially likely to feel isolated due to the limitations and difficulties caused by dialysis life. The feeling of being alone is emphasized during dry treatment sessions, which can lead to negative emotions, such as anxiety, depression, and regression. This was similarly revealed in other studies showing that hemodialysis patients experience a significant level of loneliness, and that higher levels of loneliness are associated with more symptoms of depression, impaired family functioning, and social isolation [22, 29]. However, they also appeared to overcome loneliness and strive for the future through family support communication, and independent efforts at the hospital.

In particular, middle-aged hemodialysis patients experience a sense of loss due to the necessity of repeated treatments and the unpredictability of their health conditions. This makes it challenging for them to reconstruct the meaning of their lives. They experience ambivalence about death, which further highlights the necessity of psychological support. They require tailored counseling and education programs that consider their psychological characteristics and support to transform extreme pessimism into positivity [30].

The issue of loneliness is not exclusive to that undergoing hemodialysis. Similarly, patients undergoing peritoneal dialysis may also be susceptible to experiencing comparable sentiments of loneliness. Peritoneal dialysis is a treatment modality that has been demonstrated to reduce the frequency of hospital visits and enhance patient autonomy, thereby improving QOL. However, the self-management required by this form of treatment may result in peritoneal dialysis patients experiencing heightened social isolation and the burden of care [31]. Cuevas-Budhart et al. [31] have reported that while peritoneal dialysis patients benefit from the positive effects of increased autonomy in their treatment environment, the reduction in social interactions emphasizes the growing need for psychological support. It is evident that there are significant discrepancies between the underlying causes and the coping mechanisms of loneliness exhibited by patients undergoing either hemodialysis or peritoneal dialysis. Patients undergoing hemodialysis frequently require emotional support derived from hospital-based treatments. In contrast, patients undergoing peritoneal dialysis require the implementation of support systems that can effectively mitigate the social isolation that is often a consequence of home-based treatment processes. These differences highlight the necessity for a comprehensive examination of the psychological well-being and emotional health impacts that are unique to each patient group. It is therefore imperative that comparative studies be conducted to examine the experiences of loneliness between these two patient groups. Such research provides crucial foundational data for the development of bespoke nursing strategies and intervention plans that are tailored to the specific needs of each group. Such comparative studies can enhance our understanding of the unique psychological experiences and coping mechanisms associated with different treatment modalities, thereby contributing to the advancement of care practices that address the individual needs of dialysis patients. Furthermore, they provide a crucial foundation for the development of patient-centered nursing strategies and the improvement of the overall QOL of dialysis patients.

The development of a social support network is of paramount importance for patients undergoing hemodialysis or peritoneal dialysis. Dialysis clinics and social support centers must collaborate to devise strategies that facilitate the reintegration of patients into society. This may entail the formation and nurturing of patient support groups and the implementation of referral systems for counseling. Additionally, there is a need to minimize avoidable hospitalizations and enhance QOL through programs that bolster patients' emotional well-being [31].

Family support is a relevant aspect that directly affects the QOL of hemodialysis patients [32]. Middle-aged hemodialysis patients feel a lack of understanding and communication in their relationships with their families and sometimes experience loneliness that they have to endure alone. This appeared to be the result of a lack of communication between family members and a lack of understanding of hemodialysis. Therefore, a program that not only educates families about the physical symptoms of hemodialysis but also promotes communication and understanding from an emotional perspective is needed.

The experience of social stigma can intensify feelings of loneliness among patients, which in turn impairs their capacity to engage in economic activity and maintain employment. Such isolation can result in patients perceiving themselves negatively and fearing the judgment of others, which may ultimately lead to a loss of motivation to live. It is therefore recommended that hemodialysis hospitals and social support centers implement a support system with the objective of reducing social stigma and enabling patients to actively participate in social activities. It is recommended that ongoing counseling be made available to assist patients in releasing accumulated emotions and in feeling respected for their desire for a healthy life.

Lastly, many studies have been conducted on hemodialysis patients, targeting the elderly, who make up the majority of the overall diseased population. However, health in old age is the result of progress from middle age, and just as one can have a successful old age only when one spends middle age well [21], middle-aged people who have to live with hemodialysis until death can expect a successful life on hemodialysis in old age only if they adapt well to this period. Therefore, more diverse research targeting middle-aged hemodialysis patients should be conducted to prevent problems that may persist into old age. It is believed that the answers to problems that cannot be found among the problems currently experienced by elderly hemodialysis patients can be found in middle-aged hemodialysis patients.

This study provides an in-depth understanding of the experience of loneliness among middle-aged patients on hemodialysis. It is important to note that this is not simply a negative aspect of their experience; rather, it involves a process of adaptation and meaning reconstruction. This highlights the necessity for a comparative analysis of emotional experiences between hemodialysis and peritoneal dialysis patients, with the aim of developing nursing strategies and support systems that reflect their differentiated needs. Such research will provide a crucial foundation for enhancing the QOL of patients undergoing renal replacement therapy and for promoting the overall well-being of dialysis patients from middle to old age, thereby reinforcing patient-centered nursing practice.

### 4.1. Study Limitations

Because this study targeted middle-aged patients at some hemodialysis treatment centers in Seoul, it is difficult to interpret or generalize it to all dialysis patients. Additionally, the failure to sufficiently consider diverse and special environments may also be a limitation of this study.

## 5. Conclusions

Middle-aged hemodialysis patients experience loneliness and anxiety due to the financial burden of repeated dialysis treatments and associated costs. This causes their personal lives to feel like shackles, and they suffer deeper loneliness due to a lack of communication with family and social prejudice. While patients try to cope with loneliness as part of the process of independence and adaptation, they find it difficult to derive meaning from life due to the limitations and losses in their daily lives. However, they make efforts to overcome loneliness through family support, and this experience of loneliness recurs in their individual, family, and social relationships. This study aims to explore the experiences of loneliness and loss among middle-aged hemodialysis patients and provide valuable insights for healthcare professionals to better support these patients. Additionally, the findings may contribute to future research by identifying factors related to loneliness in middle-aged hemodialysis patients.

## 6. Implications

### 6.1. Nursing Management

This study demonstrates that the loneliness and sense of loss experienced by middle-aged hemodialysis patients have significant implications for nursing management. It is essential for nursing managers to understand the emotional needs of these patients and develop targeted programs and services. For instance, integrating programs that alleviate social isolation and provide emotional support, as well as promoting communication with family members, is crucial in nursing practice. In terms of patient care, loneliness and loss can adversely affect treatment adherence and QOL. Therefore, nurses must implement tailored strategies, such as offering psychological counseling or emotional support groups, to help patients overcome loneliness and actively engage in their treatment. From a policy perspective, establishing social and emotional support systems is necessary to mitigate the effects of loneliness and loss among middle-aged hemodialysis patients. This includes developing hospital-based support networks, expanding psychological counseling services, and encouraging patients' participation in social activities. Additionally, enhancing nurse education is critical to ensure that healthcare professionals can effectively address the emotional needs of these patients, leading to more comprehensive, patient-centered care.

In conclusion, this study provides valuable insights for nursing practice, patient care strategies, policy development, and nursing education, ultimately contributing to the improvement of the QOL for middle-aged hemodialysis patients.

### 6.2. Research

The results of this study can be used to understand the experience of loneliness in middle-aged hemodialysis patients and to develop practical approaches to support psychological adjustment. Further research is needed to develop psychological and emotional support programs and to strengthen social support systems. In addition, longitudinal studies analyzing changes in the experience of loneliness and comparative studies with different kidney disease populations will allow for more comprehensive nursing and psychological intervention strategies.

## Figures and Tables

**Table 1:** General characteristics of the study participants (*n* = 11).

Participants	Age	Gender	Dialysis maintenance period (year)^∗^	Marriage	Cohabiting family	Job
1	58	Female	8	Yes	Yes	No
2	59	Female	18	Yes	Yes	Part-time job
3	54	Male	7	No	No	No
4	62	Female	21	Yes	Yes	No
5	48	Female	7	Yes	Yes	Part-time job
6	55	Female	2	No	Yes	No
7	55	Male	2	Yes	Yes	Business
8	45	Female	8	Yes	Yes	Full-time job
9	52	Female	11	Yes	Yes	No
10	62	Male	5	Yes	Yes	Business
11	57	Male	7	Yes	Yes	No

^∗^As of June 2021.

**Table 2:** Components of loneliness experience in middle-aged hemodialysis patients.

Components	Subcomponents
The loneliness felt in a life tied to dialysis like shackles	- Deep sense of loss and loneliness stemming from the constraints of dialysis - Feeling of ostracism due to difficulty participating in social activities because of illness - Endless emptiness and loneliness felt while confined in a hospital environment - Loneliness in old age hindered by reality due to chronic illness

The sorrow and loneliness of my irretrievable life	- Longing and loneliness for the days before dialysis - Disappointment and estrangement in a life not going as planned - Solitude and loneliness felt looking at oneself significantly changed from the past

Helplessness in death and isolation at the edge of life	- Deep despair and loneliness to the extent of thinking about death - Existential isolation felt from losing the meaning of life

Living everyday wrapped in solitude	- Sense of isolation in illness that must be endured alone - Living well within the lonely confines of illness

Complex emotions and alienation within the family	- Alienation stemming from mixed feelings toward the family - Loneliness felt from the inability to share emotions - Powerlessness and estrangement felt from the inability to be helpful as a family member

Lonely life in the shadow of illness and societal prejudice	- Continuous sense of exclusion felt from always being perceived as weak - Loneliness arising from limited work opportunities - Isolation and alienation stemming from societal prejudices - Loneliness and estrangement deepening as the perception of being alone intensifies

Struggling to break free from the abyss of loneliness	- Efforts to overcome loneliness with the support of family - Attempts to reduce loneliness through meaningful communication and new activities

## Data Availability

The data that support the findings of this study are available from the corresponding author upon reasonable request.
